# Transforaminal Ligaments of the Lumbar Spine: A Comprehensive Review

**DOI:** 10.7759/cureus.811

**Published:** 2016-10-02

**Authors:** Randle Umeh, Christian Fisahn, Brittni Burgess, Joe Iwanaga, Marc Moisi, Rod J Oskouian, R. Shane Tubbs

**Affiliations:** 1 Department of Anatomical Sciences, St. George's University School of Medicine, Grenada, West Indies; 2 Orthopedic Surgery, Swedish Neuroscience Institute, Seattle, USA; 3 Seattle Science Foundation, Seattle, USA; 4 Medical Education and Simulation, Seattle Science Foundation, Seattle, USA; 5 Neurosurgery, Seattle Science Foundation, Seattle, USA; 6 Neurosurgery, Swedish Neuroscience Institute, Seattle, USA

**Keywords:** review, transforaminal ligaments, spine, radicular pain

## Abstract

Once considered anomalous structures, transforaminal ligaments are not widely known and the criteria for identifying and classifying them are not universal. They are, however, of potential importance during neurological procedures, as their entrapment might lead to radicular pain.

Transforaminal ligaments are not present in all patients, but when they are, the incidence of all types of ligaments is significantly higher, with the most common type being the superior corporotransverse ligament. By diminishing the overall amount of space available for the spinal nerve to pass, many early studies concluded that transforaminal ligaments were the cause of nerve root entrapment, resulting in radicular pain. However, more recent studies conducted have claimed that the ligaments do not cause radicular pain but rather are more for the protection of nerves and vessels.

The contribution of transforaminal ligaments to radicular pain is still a topic of debate, but their role in the protection of nerves and vessels is certain. The clinician who performs interventional procedures directed toward the intervertebral foramen and the surgeon operating in this region should have a good working knowledge of the anatomy and proposed functions of the transforaminal ligaments.

## Introduction and background

Once considered anomalous structures, transforaminal ligaments are not widely known and the criteria for identifying and classifying them are not universal. There are five major classifications of transforaminal ligaments: the superior and inferior corporotransverse ligaments, the superior and inferior transforaminal ligaments, and the mid-transforaminal ligament [1-3]. Each type is of importance during surgery near the intervertebral foramen, as inadvertent traction on these structures might lead to radicular pain. Having a comprehensive understanding of the transforaminal ligaments could help better localize a patient’s source of radicular pain.

## Review

**Figure 1:**
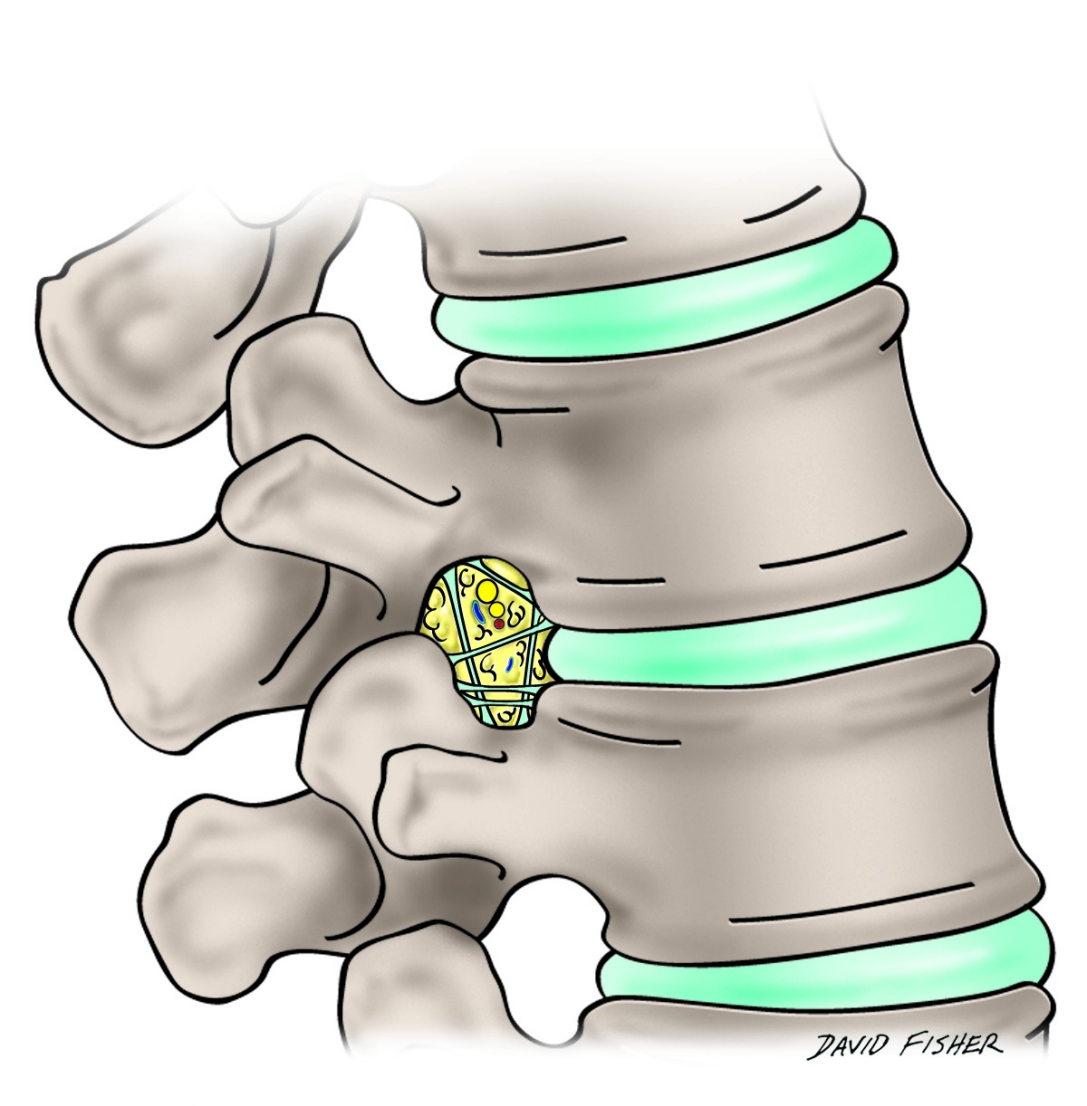
Schematic drawing of the varieties of transforaminal ligaments in the lumbar intervertebral foramen.

The intervertebral foramina allow for the passage of numerous structures, including the root of each spinal nerve, segmental spinal arteries and veins, lymphatics, and recurrent meningeal nerves [2, 4] (Figure 1).

Two broad varieties of ligaments have been found in association with the intervertebral foramina: radiating and transforaminal ligaments [5]. The latter pass through the foramina and are of particular interest as they might contribute to neurovascular compression or exacerbate intervertebral foraminal stenosis.

**Figure 2:**
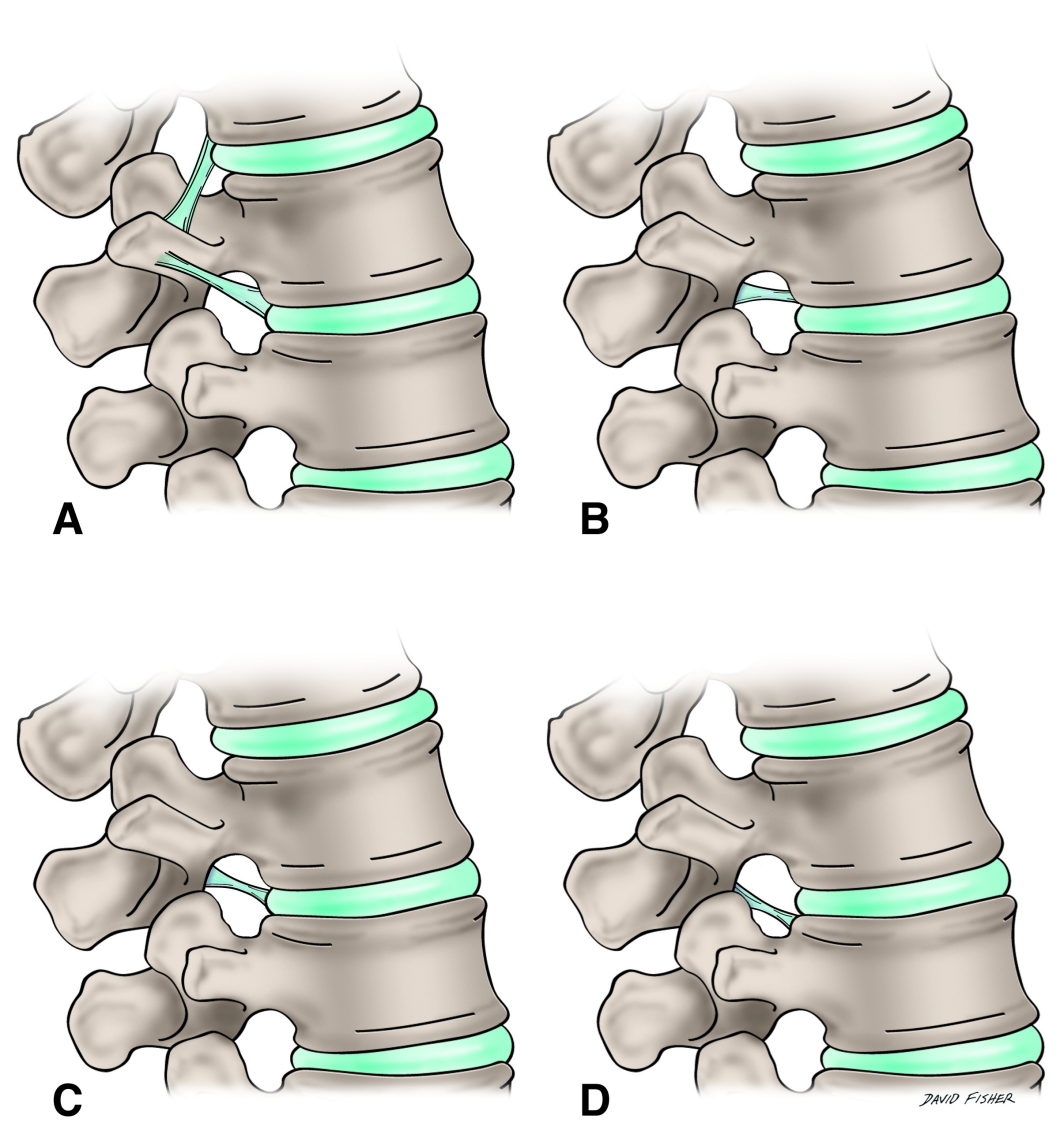
The various transforaminal ligaments as described by Golub and Silverman in 1969.

Within the boundary of each intervertebral foramen is a network of ligaments dividing the outlet into multiple subcompartments that contain their own specific anatomical structures [2]. In 1832, the French physician and anatomist, Jean-Baptiste Marc Bougery, illustrated these ligaments as traversing the intervertebral foramen [2]. These ligamentous bands were also noted in the works of Larmon and Magnuson as spanning the intervertebral foramen of the L5 segment [6-7]. However, it was Golub and Silverman [3] in 1969 who reported the presence of ligamentous bands traversing the foramina at all lumbar levels in 10 cadaveric lumbar spines (Figure 2).

Upon examination of these 10 cadaveric lumbar spines, Golub and Silverman [3] reported that nine of them showed ligamentous bands crossing the intervertebral foramina but that these bands were not seen at all levels or on both sides of the spine. They concluded that the ligaments are not always present in the lumbar spine, but when they are present, they are commonly found at L1-L2 [1, 8-9]. Bachop and Hilgendorf [1] and others supported the findings of Golub and Silverman [3] but emphasized that the ligaments were more commonly found at the level of the fifth lumbar foramen [8].

The transforaminal (intervertebral foraminal) ligaments are not widely known and were once considered anomalous structures after they had been described in the anatomical study by Golub and Silverman [8-10]. However, further, more detailed studies indicated that they were normal anatomical, rather than pathological, structures [8-10]. Min et al. found transforaminal ligaments in approximately 70-90% of human lumbar foramina [5, 10-11], although the percentage reported in the literature ranges from 17.8 to 100% since the actual criteria for identifying and classifying the transforaminal ligaments differ among studies [12].

In their pioneering study on these ligaments in 1969, Golub and Silverman [3] described the transforaminal ligaments as condensations in the fascia that overlie the exits of the intervertebral foramina and that they have ligamentous features. Besides resembling condensations of fascia more than ligaments, these structures are also less ligament-like because, with one exception, they do not connect separate bones [1-2]. The five major types of transforaminal ligaments were identified in relation to their attachments and direction [2, 11]. The obliquely running bands were designated as the superior and inferior corporotransverse ligaments while those that ran transversely were named the transforaminal ligaments [9].

The two corporotransverse ligaments are mostly distributed in the L5-S1 intervertebral foramen: the superior corporotransverse ligament attaches from the posterolateral corner of the vertebral body to the accessory process of the transverse process of the same vertebra; the inferior corporotransverse ligament connects the same posterolateral corner of a vertebral body to the transverse process below [13]. The transforaminal ligaments are more superior in the L1-L4 intervertebral foramina [13], the superior transforaminal ligaments attaching to the inferior vertebral notches, and the inferior to the superior vertebral notches; the mid-transforaminal ligaments traverse from the posterolateral corner of an annulus fibrosus to the ligamentum flavum behind and the zygapophysial joint capsule.

It must be emphasized that transforaminal ligaments are not always present, but when they are, the overall incidence of all types of these ligaments is approximately 47% [2].  The most common type is the superior corporotransverse ligament at 27% and the only ligament type of connecting separate bones is the inferior corporotransverse ligament [2]. 

The anatomical location of the transforaminal ligaments led many early studies to conclude that these condensations of fascia were the cause of nerve root entrapment resulting in radicular pain since they diminished the space available for the spinal nerve to pass [2, 5, 8-10, 12]. It has been reported that the cross-sectional area of a foramen is decreased by as much as 30% by these bands of fascia [5, 10, 12]. However, Kuofi, et al. [8] mapped the ligaments topographically, establishing their consistent presence in the foramina, and concluded, in contradiction to earlier studies, that they did not cause nerve root entrapment leading to radicular pain. Rather, they were more protective of nerves and vessels [2, 13]. Clearly, further investigation into these structures is warranted.

## Conclusions

This was a comprehensive literature review conducted on the transforaminal ligaments regarding their anatomy and function. Although there have been limited studies of their form and function, the transforaminal ligaments are believed by some to be protective of nerves and vessels traversing the intervertebral foramen while being the cause of radicular pain is still a matter of debate.
